# Co-Occurrence of *Stromatinia cepivora* and *Fusarium proliferatum* Fungi on Garlic: *In Vitro* Investigation of Pathogen–Pathogen Interactions and *In Planta* Screening for Resistance of Garlic Cultivars

**DOI:** 10.3390/plants14030440

**Published:** 2025-02-02

**Authors:** Samara Ounis, György Turóczi, József Kiss

**Affiliations:** Department of Integrated Plant Protection, Institute of Plant Protection, Hungarian University of Agriculture and Life Sciences, H 2100 Gödöllő, Hungary; ounis.samara@phd.uni-mate.hu (S.O.); turoczi.gyorgy@uni-mate.hu (G.T.)

**Keywords:** garlic, cultivars, *Fusarium proliferatum*, *Stromatinia cepivora*, resistance

## Abstract

Garlic (*Allium sativum* L.), a vital global crop, suffers significant losses from soil-borne fungal pathogens such as *Fusarium proliferatum*, responsible for *Fusarium* bulb rot, and *Stromatinia cepivora*, the causal agent of white rot. In May 2023, garlic fields near Makó City, Hungary, showed simultaneous yellowing and wilting symptoms of unknown fungal infestations, which appeared sporadically. The causal pathogens were later confirmed as *F. proliferatum* and *S. cepivora* through sampling of symptomatic garlic plants, incubation in humid chambers to stimulate fungal growth, and culturing on Potato Dextrose Agar (PDA) under sterile conditions. This was followed by hyphal tip isolation and purification. Molecular identification was performed using ITS1-2 sequencing, supported with morphological identification based on colony and microscopic features. This research aimed to elucidate pathogen interaction dynamics and assess the resistance of eleven garlic cultivars to both single and simultaneous inoculations by these pathogens, under *in vitro* and *in planta* tests. Dual culture assays of *F. proliferatum* and *S. cepivora* were studied at two time points: Day 8, marking the cessation of growth along the interacting fronts due to competitive coexistence, and Day 14, when single cultures reached full radial growth. On Day 8, inhibition percentages were 8.47% for *F. proliferatum* and 6.40% for *S. cepivora*, reflecting the initial effects of competitive interactions at the point of contact. By Day 14, inhibition rates increased to 25.39% and 28.61%, respectively, highlighting the cumulative effects of sustained competition and the growing difference between single and dual culture growth. Inoculation trials, involving placing fungal disks onto the basal areas of wounded garlic cloves, revealed considerable variability in disease incidence and severity. Cultivars such as ‘Aulxito’, ‘Sabadrome’, ‘Arno’, ‘Garcua’, and ‘Makói Tavaszi’ were highly susceptible to both pathogens, while ‘Flavor’ and ‘Sabagold’ exhibited only mild symptoms when inoculated with *F. proliferatum* and *S. cepivora*, respectively. Simultaneous inoculation resulted in more rapid and severe infections, exhibiting disease incidences above 96.00% across all cultivars. Remarkably, the cultivar ‘Elephant’ exhibited complete resistance to both pathogens, even under simultaneous inoculation, highlighting its potential for future garlic resistance breeding programs.

## 1. Introduction

Garlic (*Allium sativum* L.) is one of the most important vegetables and spice crops. It is renowned for its extensive use in cuisines globally and its medicinal properties, attributed to compounds like allicin [1]. The global cultivation of garlic, alongside onion, underscores its economic and nutritional value with an annual production exceeding 28 million tons in 2021, with China leading in production, followed by India [2]. Although the crop is largely cultivated for its cloves for culinary use, it also exhibits a broad spectrum of human health benefits, including antimicrobial, anticancer, and antioxidant activities [3]. When extracted and isolated, the bioactive compounds of garlic show a wide range of beneficial human health effects to treat various infectious diseases, non-communicable diseases, as well as metabolic and genetic disorders [1]. Beyond culinary and medicinal applications, garlic’s allelochemicals show promise as sustainable agricultural biostimulants, enhancing crop quality and resistance against pathogens [4].

During a field investigation conducted in May 2023 in the Makó region of Csongrád-Csanád County, Hungary (46.2185° N, 20.5299° E)—an area renowned for its significant role in Allium crop production—growers observed that autumn garlic plants exhibited symptoms of yellowing and wilting. These symptoms were not uniformly distributed but appeared sporadically over various fields, indicating the possible influence of a soil-borne pathogen. Detailed examination of the affected plants revealed early stages of possibly multiple soil-borne fungal infections, with characteristic symptoms of white rot and *Fusarium* bulb rot co-occurring.

*Stromatinia cepivora* (Berkeley) Whetzel (anamorpha *Sclerotium cepivorum*) is an economically significant pathogen that causes white rot disease in garlic and other Allium species [5]. This disease was first reported in Hungary in 2011 [6]. *Stromatinia cepivora* is a major limiting pathogen for onion and garlic production worldwide due to its ability to cause large yield and quality losses [7,8]. This pathogen is distinguished by its sclerotia, which can survive in soil for several years, thereby limiting the use of infected fields in a rotation system [7,8,9]. These sclerotia germinate in response to volatile organic compounds from Allium root exudates, initiating infection [10]. The symptoms of white rot typically appear from mid-season to harvest, starting with yellowing and wilting of the leaves, followed by white mycelial growth on the bulb surface or at the crown of seedlings [11]. As the infestation progresses, the root system is destroyed, leading to plant collapse. Infected bulbs often exhibit white, fluffy fungal growth, resembling cotton wool, with small black sclerotia interspersed [12]. The pathogen’s resilience and long dormancy of the sclerotia, enabling them to persist in the soil until a suitable host becomes available, make it extremely difficult to manage [9].

The *Fusarium* genus comprises a diverse group of fungal pathogens that are globally distributed and responsible for economically significant losses in a wide range of crops. Several species within this genus, such as *Fusarium oxysporum*, *F. proliferatum*, *F. culmorum*, and *F. solani*, are known to cause substantial damage to garlic, leading to symptoms such as basal plate rot and other decay types in both field-grown and stored crops [13,14,15]. Among these species, *F. proliferatum* (Matsushima) Nirenberg is of particular concern due to its widespread occurrence and significant impact on *Allium* crops production [16]. It has been associated with bulb rot in garlic, causing substantial economic losses, particularly during post-harvest storage [17,18]. *Fusarium proliferatum* infects a broad range of hosts across various climate zones, including other *Allium* crops like onions and leeks, demonstrating its capacity for adaptation [19,20]. The pathogen was first reported as the causal agent of garlic bulb rot in 1999 in Hungary during winter storage [21]. Since then, *F. proliferatum* has been identified in garlic crops across multiple regions, including Germany [22], Serbia [23], Spain [20], and Italy [17]. The symptoms of garlic rot caused by *F. proliferatum* include dry, brown necrotic spots that develop on the surface of the cloves and can progress inward. In severe cases, white mycelium and water-soaked lesions can also occur [17,24]. The pathogen often colonizes garlic roots during the growing season and remains latent, causing substantial post-harvest losses as rot develops in storage [16,17,18], where conditions such as temperature significantly influence the progression of the disease [25]. Furthermore, *F. proliferatum* is a significant mycotoxigenic species, producing toxins such as fumonisins (FB1, FB2, and FB3), moniliformin, beauvericin, fusaric acid, and fusaproliferin, all of which present a high risk to food safety [26,27].

Chemical control, while initially effective against *Fusarium* bulb rot and white rot, now faces challenges from emerging pathogen resistance and significant application costs, raising environmental and health concerns [28,29]. Despite the efficacity of biological control like the use of *Trichoderma* spp. in managing these pathogens [12], it does not offer long-term management. Consequently, developing genetically resistant cultivars has been recognized as the most sustainable strategy for managing soil-borne diseases [30,31,32].

The simultaneous occurrence of multiple soil-borne pathogens complicates disease management in agricultural settings. It is well-documented that simultaneous infections by multiple pathogens are a common phenomenon in agriculture [33,34]. Previous research has shown that host plants infected by multiple pathogens experience more severe disease impacts than those affected by a single pathogen [30,34,35]. Given that *F. proliferatum* and *S. cepivora* were observed co-occurring, this study hypothesizes that their interactions could influence infection dynamics and disease progression and seeks to investigate their interaction dynamics under controlled *in vitro* conditions. Dual culture assays were employed to determine the type of interaction these pathogens exhibit, providing insights into their ecological relationships and implications for garlic disease development. Understanding these interactions is essential for improving resistance screening methodologies and developing effective disease management strategies against multi-pathogen infestations.

Despite advances in breeding for single-pathogen resistance, such resistances often fail under field conditions where co-infections are common [30,33]. This study addresses the critical gap in understanding the interactions between *F. proliferatum* and *S. cepivora*, aiming to develop more effective, economically viable, and environmentally sustainable disease management strategies through multi-pathogen resistant cultivars [30,31,32]. The exploration of these pathogen interactions is unprecedented and essential for advancing garlic breeding programs.

Therefore, in the present work, we aim to (1) accurately identify the pathogenic fungi behind the *Fusarium* bulb rot and white rot; (2) investigate their co-occurrence dynamics and the nature of their pathogen–pathogen interactions; and (3) assess both the resistance of eleven different garlic cultivars to single infections against each pathogen, as well as their resistance to the simultaneous occurrence of *F. proliferatum* and *S. cepivora* under *in planta* conditions.

## 2. Materials and Methods

### 2.1. Isolation and Identification of the Phytopathogens

**Figure 1:**
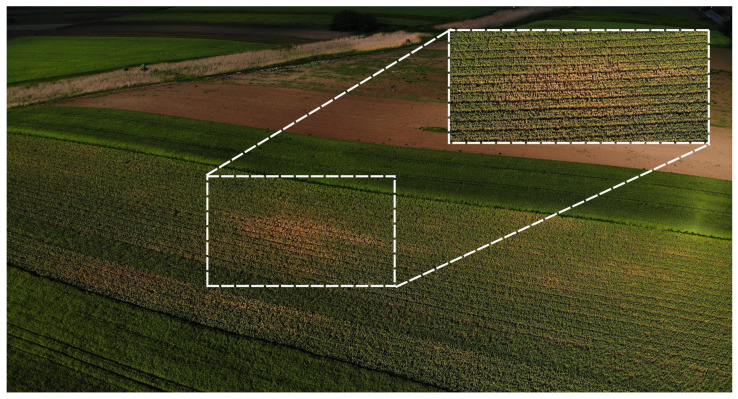
Drone image of a garlic field in Makó, from which symptomatic garlic samples were collected for this study. The visible yellowing spots, indicative of potential fungal infection, are highlighted in the image, with a zoomed-in view of one of the affected areas.

Diseased garlic plants showing fungal infection symptoms consisting of yellowing and wilting were collected from garlic fields located in the Makó region, Csongrád-Csanád county, south-east Hungary (46.2185° N, 20.5299° E). The patchy distribution of symptoms, characterized by areas of yellowing and wilting, was observed from above using drone imaging and is displayed in Figure 1.

Garlic bulbs were placed in a humid chamber to enhance fungal growth for 24 h. Sampled cloves were cut into small fragments, inoculated under sterile conditions in Petri dishes on PDA media (Potato Dextrose Agar), and incubated at 25 °C for 5 days. The developed fungal cultures were purified using hyphal tip isolation techniques [36]. The identification of the isolated fungal species was performed based on both morphological characteristics (colony morphology on different media and microscopical morphology). Fungal isolates were further identified using sequence analysis of the internal transcribed spacer (ITS1-2) region of ribosomal DNA. DNA extraction, amplification, and sequencing were conducted following SOP-115-11 protocols. The amplified sequences were analyzed using the NCBI BLAST algorithm for comparison. The molecular identification was commissioned by the authors and conducted by the Eurofins BIOMI laboratory. The obtained pure cultures were stored at 5 °C on PDA slants to maintain viability for further studies.

### 2.2. Selective Media Tests

To investigate selective media that promote the fungal growth of the isolated pathogens, pure fungal colonies were subcultured on Potato Dextrose Agar, Sabouraud Dextrose Agar, Czapek-Dox Agar, and Malt Extract Agar. All plates were incubated at 25 °C and observed regularly for the following 10 days.

### 2.3. In Vitro F. proliferatum and S. cepivora Interaction Test

In order to assess the interaction between the two phytopathogens, a dual culture test was first employed. On the selected medium suitable for both pathogens (Sabouraud Dextrose Agar), fungal disks measuring 7 mm from 8-day-old cultures were positioned on opposite sides of the Petri dishes. As for the control plates, fungal disks containing the pathogens separately were placed peripherally to assure consistency. The plates were then incubated at a constant temperature of 25 °C. To minimize variability across plates, all fungal plugs were taken from the actively growing margins of cultures grown under identical conditions. Three independent experiments were performed, each with 4 replicates per treatment group. The number of replicates was chosen to ensure sufficient representation of fungal interactions and to achieve a robust effect size, as measured by eta-squared (η^2^), confirming the reliability of the observed differences between treatments. The controlled nature of the *in vitro* environment minimized variability and facilitated precise measurement of fungal growth parameters. Colony growth was monitored daily, with radial growth measurements recorded as the means of two perpendicular diameters across each fungal colony.

To assess the interaction between the two pathogens, the interaction strength for each pathogen is calculated as follows and is assessed at two time points: the day of contact and the day of full growth of single cultures:

- Growth inhibition of *F. proliferatum* by *S. cepivora*:

*F. proliferatum* growth inhibition (%) = (Cf − Df)/Cf × 100, (1)

where Df is the distance *Fusarium* grows towards *Stromatinia* and Cf is the mean of the radial growth of the *Fusarium* control plates.

- Growth inhibition of *S. cepivora* by *F. proliferatum*:

*S. cepivora* growth inhibition (%) = (Cs − Ds)/Cs × 100, (2)

where Ds is the distance *Stromatinia* grows towards *Fusarium* and Cs is the mean of the radial growth of the *Stromatinia* control plates.

### 2.4. Resistance Against Single Infections of F. proliferatum and S. cepivora

#### 2.4.1. Garlic Cultivars Preparation

In this study, eleven garlic cultivars were selected for screening to assess their resistance against *S. cepivora* and *F. proliferatum*. The cultivars contained both autumn and spring varieties to cover a range of genetic diversity. The spring varieties evaluated were ‘Flavor’, ‘Arno’, and ‘Makói Tavaszi’. Notably, ‘Makói Tavaszi’ is the only local cultivar examined, distinguishing it from the remaining French varieties, which are cultivated globally. The autumn varieties included ‘Thermidrome’, ‘Messidor’, ‘Sabadrome’, ‘Sabagold’, ‘Aulxito’, and ‘Garcua’ and ‘Elephant’. Although commonly referred to as Elephant garlic, this cultivar is not true garlic, but rather belongs to a different species, *Allium ampeloprasum*, closely related to leeks.

#### 2.4.2. *In Planta* Inoculation of the Pathogens

For the *in planta* inoculation of *S. cepivora* and *F. proliferatum* on the selected garlic cultivars, the employed methodology was the one suggested by Esler and Coley-Smith [37]. Fungal disks, 8 mm in diameter, were cut from 10-day-old cultures of each pathogen, which had been cultivated on selective media. These disks were placed on small wounds on the basal area of the cloves and securely wrapped with parafilm. The samples were incubated at room temperature in humid chambers. Three independent experiments were conducted, each with 5 replicates per treatment group. Each replicate consisted of 5 cloves, ensuring that variability within replicates was minimized. The number of replicates was chosen to account for the higher biological variability of garlic cloves and to achieve a meaningful eta-squared value (η^2^), ensuring robust detection of significant differences in disease incidence among treatment groups. Garlic cloves were randomly assigned to treatment groups. Variability in garlic clove size and physiological state was minimized by selecting cloves of similar size and weight. Throughout the evaluation period, the development of mycelia and sclerotia and the progression of necrotic symptoms were documented and analyzed over time.

#### 2.4.3. Inspection and Analysis

To calculate the percentage of disease incidence for both pathogens, the following formula provided by Manandhar et al. [38] was employed:

Disease incidence (DI (%)) = Number of diseased samples/Total number of samples × 100

The disease severity on various garlic cultivars was systematically assessed and classified based on visual evaluations and the severity of symptoms manifested on the cloves of the inoculated replicates. The pathogenicity was categorized under the 5 following classes outlined by Mondani et al. [39]:

- Class 0: cloves were completely asymptomatic, showing no signs of infection (0%).
- Class 1: cloves exhibited small brown spots localized near the basal plate (10%).
- Class 2: moderate infection, with brown spots covering half of the basal plate (35%).
- Class 3: more extensive infection, with brown spots on the whole perimeter of the basal plate, occasionally accompanied by mycelial growth and/or sclerotia (65%).
- Class 4: severe infection, with brown spots on the basal plate extending to the bulb and with prominent visible mycelium and/or sclerotia (90%).

### 2.5. Resistance Against Simultaneous Infections of F. proliferatum and S. cepivora

To investigate the combined impact of *F. proliferatum* and *S. cepivora* on the various garlic cultivars, a co-inoculation approach was undertaken. The methodology employed for the dual inoculation mirrored the protocol established for individual pathogen inoculation, adhering to the methodology suggested by Esler and Coley-Smith [37]. However, in this experiment, two fungal disks—each cut from the respective 10-day-old cultures of *F. proliferatum* and *S. cepivora*—were concurrently inoculated onto the garlic cloves and were securely wrapped with parafilm. The samples were incubated at room temperature in humid chambers. Similar to the single inoculation experiments, three independent experiments were conducted, each with 5 replicates per treatment group. Each replicate consisted of 5 cloves, ensuring that variability within replicates was minimized. The number of replicates was chosen to account for the higher biological variability of garlic cloves and to achieve a meaningful eta-squared value (η^2^), ensuring robust detection of significant differences in disease incidence among treatment groups. Variability in garlic clove size and physiological state was minimized by selecting cloves of similar size and weight. The growth of mycelia and sclerotia and the occurrence of necrosis were documented through time.

The classification system suggested by Mondani, Chiusa et al. [39] employed for assessing the individual pathogenicity of *F. proliferatum* and *S. cepivora* was also applied in this test, along with the percentage of disease incidence formula provided by Manandhar, Timila et al. [38].

### 2.6. Statistical Analysis

Statistical analysis was conducted utilizing SPSS software (version 27.0). Data were subjected to analysis of variance (ANOVA) and Tukey post-hoc tests to determine the significance of differences observed within the experimental conditions at *p* < 0.05. Additionally, eta-squared values (η^2^) were calculated as a measure of effect size to quantify the proportion of variance explained by the experimental factors. The experimental design is summarized in Table 1. Detailed ANOVA results are available in Supplementary Tables.

## 3. Results

### 3.1. Isolation and Identification of the Phytopathogens

The inoculation of symptomatic parts from garlic tissues onto growth media, followed by purification protocols, facilitated the isolation of two distinct fungal pathogens.

Macroscopic identification was conducted based on colony morphological characteristics. On PDA, *F. proliferatum* colonies exhibited distinct aerial white mycelia with a dark violet pigmentation on PDA, while *S. cepivora* was identified by the abundant development of black microsclerotia within the colonies, a specific trait of the pathogen.

**Figure 2:**
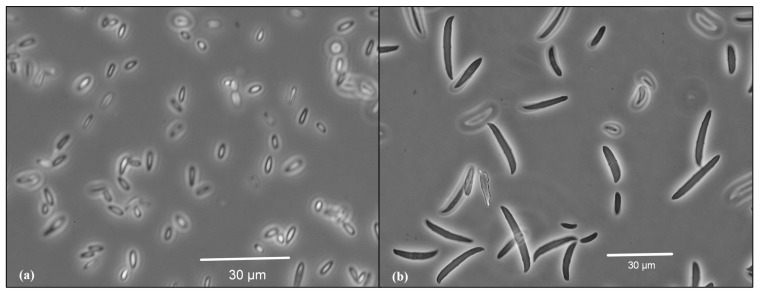
Microconidia (**a**) and macroconidia (**b**) of the *Fusarium* strain isolated from garlic.

Microscopical examination of *F. proliferatum* revealed distinct features confirming its identification (Figure 2). Under the microscope, slender, thin-walled macroconidia were observed, which were relatively straight and predominantly 3- to 5-septate. The apical cells were curved, and the basal cells appeared poorly developed. These macroconidia were found forming in pale orange sporodochia, although these structures were infrequently observed. Microconidia were abundant, forming moderate-length chains produced by both monophialides and polyphialides. They were primarily club-shaped with a flattened base. Importantly, no chlamydospores were detected, which is characteristic of this species and further corroborates its identification. Our observations align with descriptions of *F. proliferatum* morphology, as detailed by Leslie and Summerell [40].

ITS sequencing complemented these findings and provided additional confirmation. The ITS sequences were compared against the NCBI database using the BLAST algorithm. *Stromatinia cepivora* was identified with 100% similarity based on the ITS1-2 region (Accession No. KP257580.1) and showed no nucleotide polymorphisms. The second isolate was identified as part of the *Fusarium fujikuroi* species complex (FFSC) with 100% similarity. However, *F. proliferatum*—a known member of the FFSC—has previously been identified as the primary causal agent of dry rot in garlic [41]. Given this established role, the morphological observations, and the genetic similarity observed, the isolate is conclusively identified as *F. proliferatum*. These observations, encompassing macroscopic, microscopic, and molecular analyses, confirm the identity of the isolates. Phylogenetic trees, based on the results of BLAST analyses, were constructed to validate these identifications and are provided in Supplementary Figures S1 and S2.

### 3.2. Selective Media Analysis

**Figure 3:**
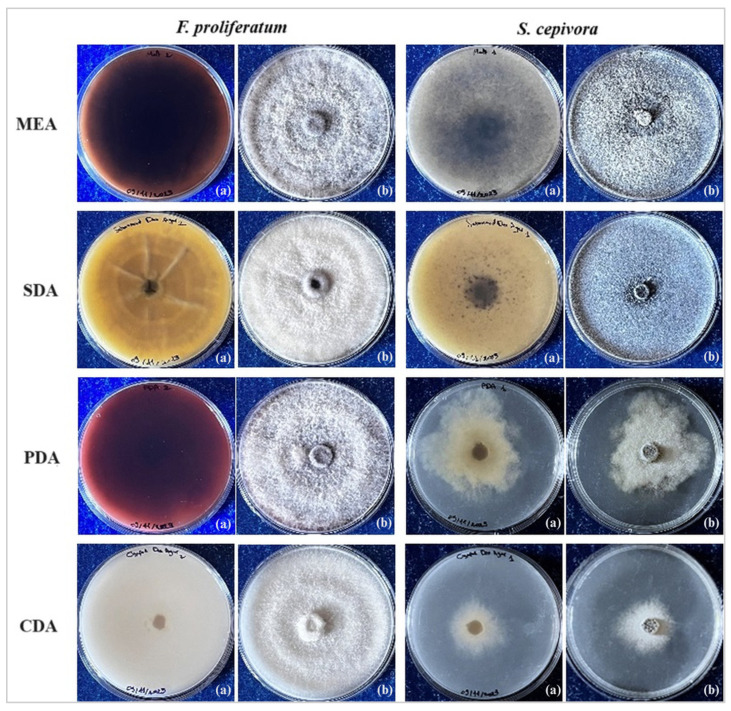
Growth phenotypes and colony morphology of *Fusarium proliferatum* and *Stromatinia cepivora* on different media: Malt Extract Agar (MEA); Sabouraud Dextrose Agar (SDA); Potato Dextrose Agar (PDA); Czapek-Dox Agar (CDA). For each medium, (**a**) presents the reverse side of the Petri dish and (**b**) presents the upper side (images taken by the authors).

Figure 3 shows the growth phenotypes and colony morphology of *F. proliferatum* and *S. cepivora* on different media. All four employed media successfully supported the growth of *Fusarium proliferatum.* Malt Extract Agar (MEA) promoted the formation of white mycelia with progressively intensifying dark red pigmentation. On Sabouraud Dextrose Agar (SDA), the fungus developed aerial white colonies accompanied by yellow to light brown pigmentation. Potato Dextrose Agar (PDA) supported the growth of white mycelia with dark violet pigmentation. In contrast, Czapek-Dox Agar (CDA) was conducive to the growth of cotton-like white mycelia exhibiting white pigmentation.

In contrast, for *S. cepivora*, only Malt Extract Agar (MEA) and Sabouraud Dextrose Agar (SDA) proved to be effective in promoting prolific fungal growth, showing yellowish and white pigmentations, respectively. Furthermore, both media facilitated the development of abundant black sclerotia on the surface of the cultures.

### 3.3. In Vitro F. proliferatum and S. cepivora Interaction Assay

**Figure 4:**
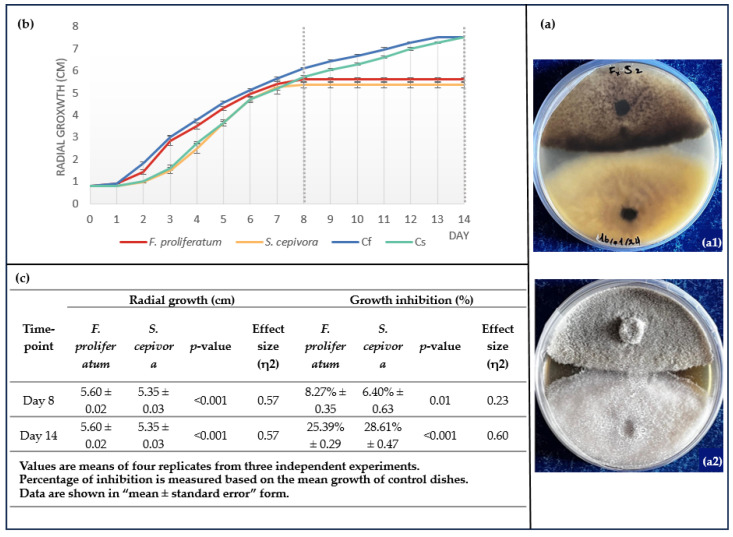
(**a**) Pathogen–pathogen interaction observed in co-culture of *S. cepivora* and *F. proliferatum* on SDA medium. Section (**a1**) shows the reverse side of the Petri dish, and section (**a2**) shows the front side (images taken by the authors). (**b**) Daily radial growth of *F. proliferatum* and *S. cepivora* in dual inoculation compared to control colonies over a 14-day period. Cf represents the radial growth of the *F. proliferatum* control plates. Cs represents the radial growth of the *S. cepivora* control plates. Vertical dashed lines indicate key time points: Day 8, when growth ceased in dual cultures, and Day 14, when single cultures reached full radial growth. Error bars represent SD values. (**c**) Radial growth and growth inhibition measurements of *Fusarium proliferatum* and *Stromatinia cepivora* under dual culture conditions on Days 8 and 14. The table presents results of statistical analyses, including *p*-values and effect sizes (eta-squared η^2^), highlighting the extent and significance of competitive interactions at each time point.

The interactions between *F. proliferatum* and *S. cepivora* were observed and quantified daily through a dual culture assay. The experiment was conducted on Sabouraud Dextrose Agar (SDA), which prompted the growth of both pathogens. Figure 4a illustrates visual observations of the dual cultured dishes of *S. cepivora* and *F. proliferatum*. Both fungi have kept to their side of the dish, neither overgrowing the other. This further illustrates the hypothesis that these two fungi have a certain type of co-existence, which is likely a competitive one. Figure 4a also highlights a clear area of contact between the two fungi, where there is a significantly pronounced darkening of the colonies of *S. cepivora*.

Daily radial growth measurements were recorded throughout the experiment and are presented in Figure 4b over a period of 14 days with a clear sectioning of the two studied time points, providing a detailed visualization of the growth dynamics over time. Figure 4c shows the radial growth pattern of each pathogen under both co-cultured and control conditions, as well as the percentage of growth inhibition presented.

Both fungi exhibited growth patterns comparable to their respective controls until approaching the proximity zone of interaction (Figure 4b). In dual cultures, fungal growth slowed earlier on the mycelial front directed toward the opposing pathogen due to competition at the proximity zone, while the mycelial front expanding away from the interaction continued to grow. Conversely, single cultures grew uninhibited, resulting in slightly greater radial growth at the same time point (Day 8), when dual cultures had already ceased growth at the point of contact between the colonies on the interacting front. *Fusarium proliferatum* exhibited a growth reduction to 5.60 cm from 6.10 cm observed under control conditions. Similarly, *S. cepivora* showed reduced growth, measuring 5.35 cm in the presence of *F. proliferatum* compared to its control growth of 5.72 cm. The percentage growth inhibition recorded for *F. proliferatum* and *S. cepivora* was 8.27% and 6.40%, respectively. Results of one-way ANOVA for the first time point (Day 8), summarized in Figure 4c, confirmed significant differences in radial growth patterns between *F. proliferatum* and *S. cepivora* under dual culture conditions, with the results being statistically significant at *p* < 0.001. The effect size (η² = 0.57) indicates a large effect, meaning a substantial proportion of the variance in radial growth can be attributed to the fungal species at this time point. For growth inhibition, ANOVA revealed a statistically significant difference at *p* = 0.01, with an effect size (η² = 0.23) representing a moderate effect, suggesting that the competitive interaction had a noticeable impact on the inhibition rates by Day 8. Detailed ANOVA results are provided in Supplementary Tables S1–S4.

On Day 14, the inhibition zone between the colonies remained stable on dual culture plates, with no further growth observed along the interacting fronts. The non-interacting fronts of both fungi had already reached their respective maximum radial extents on Day 8, indicating that resource competition and mutual inhibition had effectively halted growth. The darkening of the *S. cepivora* colonies in closest proximity to *F. proliferatum* became relatively more pronounced, suggesting an intensified response to the interaction. In the control plates, *F. proliferatum* and *S. cepivora* continued to grow, reaching full radial growth of 7.5 cm by Day 13 and Day 14, respectively. The growth inhibition at the second studied time point (Day 14) reached 25.39% for *F. proliferatum* and 28.61% for *S. cepivora*, highlighting the extent of the competitive interaction. On Day 14, one-way ANOVA confirmed continued significant differences in radial growth patterns between *F. proliferatum* and *S. cepivora* under dual culture conditions (*p* < 0.001), with a large effect size (η² = 0.57) observed at this time point. For growth inhibition, the results were highly significant (*p* < 0.001), with an effect size (η² = 0.60) indicating a large effect, reflecting that the difference in growth between the control and dual culture conditions had become more pronounced on Day 14. Detailed ANOVA results are provided in Supplementary Tables S1–S4.

**Figure 5:**
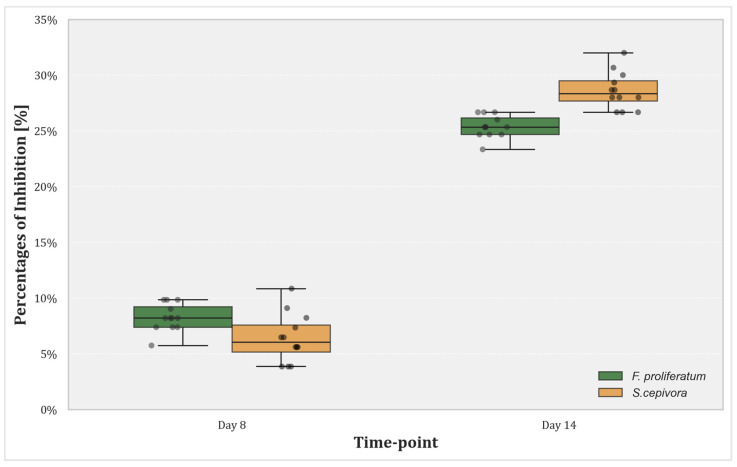
Box plot illustrating the percentage of growth inhibition of *F. proliferatum* and *S. cepivora* in dual culture conditions at two time points: Day 8 and Day 14. Day 8 represents the time when dual culture colonies ceased growth due to competitive interactions, while Day 14 corresponds to the point where single cultures reached full radial growth. The horizontal line within each box indicates the median, while the top and bottom of the boxes represent the third and first quartiles, respectively. The whiskers extend to 1.5 times the interquartile range, and jitter points represent individual data points for each time point and condition, illustrating the spread and variability of the data.

The box plot visualized in Figure 5 illustrates the percentage of growth inhibition of the pathogens under dual culture conditions at the two critical time points and further confirms the trends observed in Figure 4b. The inclusion of jitter points in this visualization provides additional insight into the distribution of individual data points across replicates.

On Day 8, growth inhibition is relatively low for both fungi, reflecting the initial effects of competitive coexistence in dual cultures. The difference between dual cultures and single cultures in terms of growth is moderate, with inhibition rates remaining consistent across replicates. By Day 14, inhibition rates increased significantly for both fungi, with *S. cepivora* showing slightly higher inhibition percentages than *F. proliferatum*. At this stage, single cultures have reached full radial growth, while dual cultures exhibit sustained growth inhibition. The visualized data highlight that the difference between dual culture and single culture growth intensifies over time, demonstrating the cumulative impact of prolonged interaction.

### 3.4. Resistance Against Single Infections of F. proliferatum and S. cepivora

**Figure 6:**
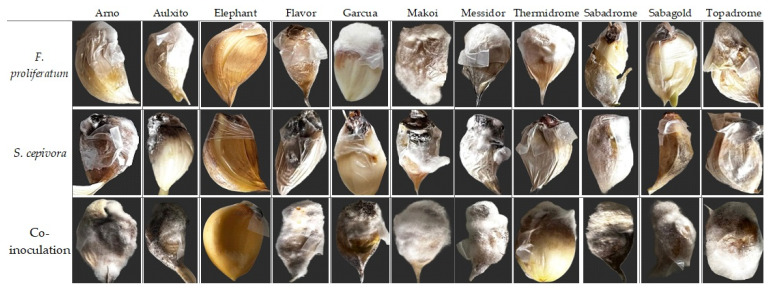
Disease symptoms of *Fusarium* bulb rot and white rot due to individual and co-inoculations with *F. proliferatum* and *S. cepivora* on the eleven tested garlic cultivars (images taken by the authors).

The pathogenic impact of *F. proliferatum* and *S. cepivora* following the *in planta* inoculation of fungal disks of the pathogens on the basal plates of the garlic cultivars was individually assessed. The evaluation of disease incidence was systematically expressed as the percentage of infected samples relative to the total number of samples per cultivar, and the severity of symptoms was classified according to the observed symptoms, which ranges from Class 0 (asymptomatic) to Class 4 (severe infection). Figure 6 shows disease symptoms of Fusarium bulb rot and white rot due to individual and co-inoculations with *F. proliferatum* and *S. cepivora* on the eleven tested garlic cultivars.

**Figure 7:**
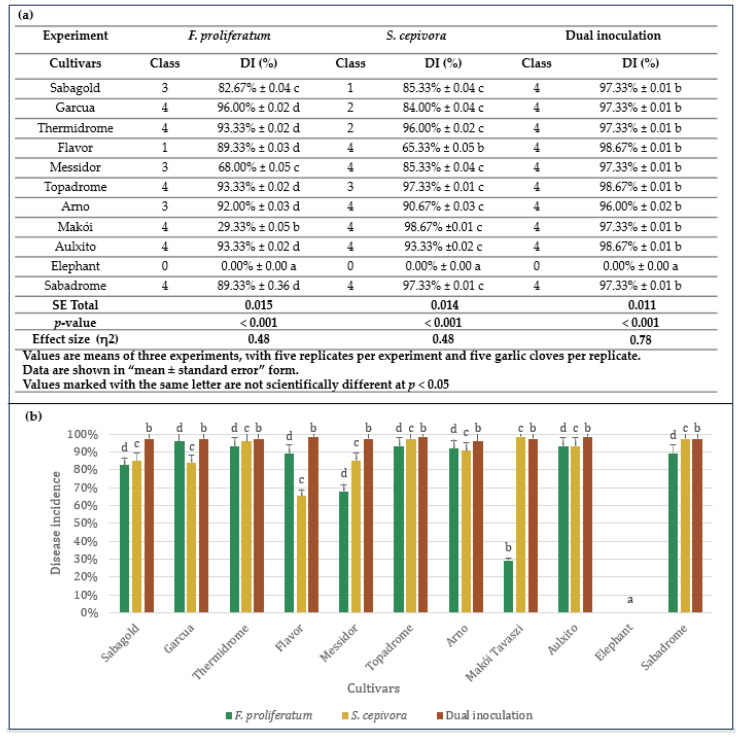
(**a**) Disease incidence and severity classification of garlic cultivars due to single and dual inoculation with *F. proliferatum* and *S. cepivora*. Statistical analysis was performed, and *p*-values and effect sizes (eta-squared η^2^) are included. (**b**) Bar chart presenting disease incidences of garlic cultivars due to single and dual inoculation with *F. proliferatum* and *S. cepivora*. The data were analyzed using Tukey’s post-hoc test, and significant differences between groups are indicated, with values marked with the same letter not being scientifically different. Error bars represent SD values.

The results summarized in Figure 7a show the class and disease incidence percentage for each cultivar under single and dual inoculation conditions. Figure 7b provides a visualization of disease incidences across garlic cultivars under these inoculation trials. Results of one-way ANOVA confirmed that the differences in disease incidence were statistically significant (*p* < 0.001) for *F. proliferatum* and *S. cepivora* single inoculations on garlic cultivars. The effect size (η^2^ = 0.48) indicates a moderate to large effect, suggesting that fungal species and their interactions with the different garlic cultivars accounted for a substantial portion of the variance in disease incidence in single inoculations. Detailed ANOVA results are provided in Supplementary Tables S5 and S6.

Initial symptoms were first observed at 5 days post-inoculation. *Fusarium proliferatum* showed severe infection (Class 4) in cultivars ‘Garcua’ (96.00%), ‘Thermidrome’ (93.33%), ‘Topadrome’ (93.33%), ‘Aulxito’ (93.33%), and ‘Makoi Tavaszi’ (29.33%). This severity was characterized by rotting of the basal plate, extensive brown spotting that extended from the basal plate to the clove, and a prominent presence of mycelium and sclerotia covering the entire clove. ‘Messidor’ (68.00%), ‘Arno’ (92.00%), and ‘Sabagold’ (82.67%) demonstrated Class 3 severity. Brown spots and discoloration covering half of the basal plate characterized this classification. ‘Flavor’ (89.33%) displayed mild infection marked by isolated brown spots on all samples and was categorized under Class 1, indicative of slight infection.

Exceptionally, ‘Elephant’ was completely symptomless, with a disease incidence of 0%, indicating the highest level of resistance among all tested cultivars.

In terms of *S. cepivora*, severe infections (Class 4) were observed in ‘Flavor’ (65.33%), ‘Messidor’ (85.33%), ‘Arno’ (90.67%), and ‘Sabadrome’ (97.33%), with symptoms including extensive browning, rotting, and mycelial growth that enveloped more than half of each clove. Similarly, ‘Makoi Tavaszi’ and ‘Aulxito’, recording 98.67% and 93.33% disease incidences, respectively, were classified under the most severe category. ‘Topadrome’ (97.33%) exhibited Class 3 severity, showing extensive infection characterized by localized mycelium formation and browning of the basal plate. ‘Garcua’ (84.00%) and ‘Thermidrome’ (96.00%) exhibited moderate infection. ‘Elephant’ remained intact against *S. cepivora*, showing no infection symptoms.

### 3.5. Resistance Against Simultaneous Infections of F. proliferatum and S. cepivora

The pathogenic effects of *F. proliferatum* and *S. cepivora* on various garlic cultivars were assessed using a dual inoculation approach under *in planta* conditions. This methodology mirrored the protocol established for individual pathogens but involved the simultaneous application of fungal discs from both pathogens onto the basal plates of garlic cloves. Disease incidence was quantified as the percentage of infected samples relative to the total number of samples per cultivar, and symptom severity was categorized on a scale from Class 0 (asymptomatic) to Class 4 (severe infection). The results summarized in Figure 7a show the class and disease incidence percentage for each cultivar under single and dual inoculation conditions, while Figure 7b provides a visualization of disease incidences across garlic cultivars. For dual inoculations, one-way ANOVA also revealed statistically significant differences (*p* < 0.001) with a larger effect size (η^2^ = 0.78). This large effect reflects the stronger and more pronounced interaction between the two fungi, as dual inoculation resulted in more severe symptoms and higher disease incidence compared to single inoculations. More detailed statistical results are available in Supplementary Tables S5 and S6.

Advanced infection symptoms, including browning at the base and mycelium formation, appeared as early as 2 days post-inoculation, indicating a rapid onset of pathogenic activity. These symptoms were followed by the appearance of sclerotia after 4 days. The co-inoculation led to severe infections (Class 4) in all cultivars except ‘Elephant’, which remained asymptomatic with a disease incidence of 0%, demonstrating its robust resistance. All ten remaining garlic cultivars recorded disease incidences above 96.00%, highlighting their high susceptibility to the combined pathogenic effects of *F. proliferatum* and *S. cepivora*. The Class 4 severity observed in these susceptible cultivars was characterized by extensive rotting at the basal plate, pronounced browning that extended from the basal plate to the cloves, and significant mycelium and sclerotia development enveloping the cloves (Figure 6). Notably, these symptoms manifested much more rapidly than in the individual inoculations of the pathogens.

## 4. Discussion

### 4.1. Dynamics of the Interaction of S. cepivora and F. proliferatum

The interaction assay of *F. proliferatum* and *S. cepivora* revealed notable dynamics within the dual cultures of the pathogens. Abdullah, Moffat et al. [34] highlighted various types of pathogen–pathogen interactions, including competition, cooperation, coexistence, and mutualism. In our study, the findings from the dual culture assay provide compelling evidence of competitive coexistence between *F. proliferatum* and *S. cepivora,* as observed at both Day 8 and Day 14. On Day 8, the fungi exhibited mutual growth limitation, with radial expansion ceasing at the interaction fronts. This early stabilization of the inhibition zone highlights the rapid establishment of competitive coexistence, driven by resource competition and spatial constraints. The interaction at this stage reflects a balance where neither fungus could overcome the inhibitory effects exerted by the other, resulting in restricted growth along the shared boundary. By Day 14, single cultures of the pathogens reached full radial growth. The continued stability of the inhibition zone, with no further growth along the interacting fronts, demonstrates the persistence of competitive coexistence. The establishment of a stable inhibition zone demonstrates that both pathogens can coexist within the same environment without one completely outcompeting the other. This interaction is indicative of mutual growth limitation, where resource competition and proximity-driven interactions prevent further radial expansion along the interacting fronts. The measurements at two distinct time points, Day 8 and Day 14, allowed us to capture both the early establishment of the inhibition zone and the cumulative effects of competitive coexistence over time. Day 8 represents the point when fungal growth in dual cultures ceased due to mutual limitation, providing insight into the onset of competitive interactions. In contrast, Day 14 reflects the sustained interaction dynamics and the long-term stabilization of the competitive equilibrium, emphasizing the persistence and extent of growth inhibition. These two time points offer a comprehensive view of how competitive coexistence evolves temporally in dual culture conditions. Yuan and Chen [42] described co-cultivation as an emerging and potential way to investigate microbial interaction under laboratory conditions, using a double-sided Petri dish to explore the interactions between *Monascus* spp. and *Aspergillus niger*. They found that the interaction between these fungi did not show antagonism but rather a symbiotic relationship, where each fungus influenced the production of secondary metabolites in the other. This finding supports our observations where both fungi coexisted without one outcompeting the other. Yuan and Chen [42] also noted that the color of the *Monascus* spp. colony that contacted *A. niger* became redder, similar to our observation where the area of *S. cepivora* in contact with *F. proliferatum* became significantly darker, likely due to the abundance of sclerotia production.

### 4.2. In Vitro Resistance Screening Against S. cepivora and F. proliferatum via Single Inoculation

The resistance screening against *S. cepivora* showed significant variability in incidence and severity among the tested garlic cultivars. ‘Messidor’, ‘Flavor’, ‘Arno’, and ‘Sabadrome’ exhibited the most severe symptoms, including advanced mycelium and sclerotia formation leading to rotting. Exceptionally, ‘Flavor’ exhibited moderate disease incidence, despite the severe symptoms it displayed, suggesting partial resistance to initial infection yet rapid progression of symptoms once the cloves are infected. ‘Topadrome’, ‘Aulxito’, and ‘Makói Tavaszi’ showed moderate symptoms, while ‘Thermidrome’ and ‘Garcua’ exhibited milder symptoms. ‘Sabagold’ showed only slight symptoms, suggesting partial resistance against white rot. In complete contrast to all other tested varieties, ‘Elephant’ displayed complete resistance, showing no symptoms of infection.

Akter et al. [43] tested eight garlic varieties and found significant variability in disease incidence and severity caused by *S. cepivora*, which is consistent with our findings where ‘Elephant’ showed complete resistance, while the other cultivars showed different levels of susceptibility. Coley-Smith and Entwistle [44] found no resistance among five garlic cultivars under field conditions. Similarly, Adams and Papavizas [45] found that resistance to *S. cepivora* was rare in the *Allium* genus, consistent with the high susceptibility seen in our study across most garlic cultivars. However, in a study conducted by Delgadillo-Sánchez et al. [46], lower susceptibility in white cultivars like ‘Perla’ and ‘Blanco de Cortazar’ was observed, which is similar to ‘Elephant’ and ‘Sabagold’ in our study, though other white cultivars showed high susceptibility.

Esler and Coley-Smith [37] suggested that resistance in Allium species might result from the inability to stimulate sclerotial germination. This suggests that the robust resistance observed in ‘Elephant’ in the current study was potentially due to non-stimulatory properties or unique genetic traits. This necessitates carrying out more detailed biochemical analyses and genetic studies to identify these traits and to investigate the genetic basis of resistance in ‘Elephant’ to identify its resistance genes.

Similar to the pathogenicity observed with *S. cepivora*, inoculations of *F. proliferatum* demonstrated substantial variability among garlic cultivars in their response to infection. Nearly all cultivars exhibited susceptibility to bulb rot disease, displaying moderate to high disease incidences. Notably, the ‘Makoi’ cultivar showed a 20% disease incidence but presented the most severe class of symptoms, highlighting the complex interaction between disease incidence and symptom severity. This cultivar demonstrated resistance to initial infection, as reflected in the low disease incidence. However, in the few cloves that became symptomatic, the pathogen’s progression was rapid and severe. This cultivar serves as an example of a cultivar that resists infection effectively but is highly vulnerable to severe damage in isolated cases of infection. These observations align with the findings of Jannatun et al. [47], who highlighted significant differences in disease incidence among eight garlic varieties infected with *F. proliferatum*. BARI Rashun-4 exhibited the highest disease incidence at 25%, while the Local Indian variety had the lowest at 3.80%. These findings underscore the variability in how *F. proliferatum* affects different garlic cultivars, suggesting that certain varieties are more susceptible to high disease incidence [47].

The severity of the disease varied significantly; all cultivars exhibited the most severe class of symptoms, except for ‘Flavor’, which displayed only slight infection symptoms, suggesting potential partial resistance despite its high incidence rates. This suggests that cultivars such as ‘Flavor’ may possess tolerance mechanisms that suppress symptom progression, limiting the pathogen’s ability to cause severe damage despite its high infection rate. Most notably, ‘Elephant’ once again exhibited complete resistance, showing no symptoms of infection and a 0% disease incidence, mirroring its reaction against *S. cepivora*. This echoes the findings of Jannatun, Fatema et al. [47], who screened eight garlic varieties for resistance to *F. proliferatum*, finding significant variation in susceptibility.

Filyushin et al. [48] investigated the genetic response of garlic cultivars to *F. proliferatum* by identifying and analyzing class I chitinase genes (AsCHI1–7). Resistant cultivars, including ‘Sarmat’, ‘Kuntsevsky’, and ‘Ershui’, exhibited higher expression of AsCHI2, AsCHI3, and AsCHI7, particularly in roots and cloves, compared to susceptible cultivars such as ‘Strelets’ and ‘Sofievsky’. This study suggests that chitinase genes could be strategically leveraged in breeding programs to enhance resistance to *F. proliferatum* [48]. This is particularly relevant to our findings, where the cultivars ‘Flavor’ and ‘Elephant’ demonstrated partial and complete resistance, respectively, to *Fusarium* bulb rot. The resistance observed in ‘Elephant’ may be attributed to genetic factors unique to its classification within *Allium ampeloprasum*, which could potentially be identified and utilized in breeding programs to enhance resistance in commercially important garlic cultivars. By exploring these genetic traits, breeding programs could develop new varieties with enhanced resistance to both white rot and *Fusarium* bulb rot. Such an approach holds promise for significantly reducing the occurrence and impact of these diseases, aligning with sustainable agriculture goals by minimizing dependency on chemical controls. Furthermore, leveraging ‘Elephant’ as a genetic resource opens new possibilities for durable, species-crossing resistance mechanisms that could strengthen the resistance of garlic plants against a broad range of pathogens.

### 4.3. In Vitro Resistance Screening Against the Dual Inoculation of F. proliferatum and S. cepivora on Garlic Cultivars

Despite the competitive coexistence of the fungi observed *in vitro*, the dual inoculation on garlic cultivars resulted in significantly increased disease severity compared to individual inoculations. All tested cultivars, except for ‘Elephant’, exhibited disease incidences above 96.00%, with symptoms such as rotting and mycelial formation appearing three days earlier than in single infections, developing into much more severe symptoms that envelop the whole clove. This confirms that while the pathogens exhibit competitive coexistence *in vitro*, their combined presence *in planta* can lead to synergistic effects that exacerbate disease symptoms. The competitive coexistence observed in the dual culture assay, where mutual inhibition limited radial growth, reflects the ability of the pathogens to compete for limited resources in an *in vitro* controlled environment. However, *in planta*, the pathogens interact differently due to host factors such as the availability of diverse nutrients, structural barriers, and host metabolic responses. These conditions enable a synergistic relationship, wherein the activity of one pathogen facilitates the colonization or pathogenicity of the other. This shift is evident in the earlier onset of symptoms and the significantly more severe manifestations under dual inoculations compared to single inoculations.

Fang et al. [49] examined the effects of co-infection by *Fusarium oxysporum* f. sp. *medicaginis* and *Rhizoctonia solani* on diverse alfalfa varieties. Their findings showed that co-infection led to significantly increased disease severity and reductions in plant growth and biomass allocation compared to single infections. This mirrors our results in garlic, where co-inoculation resulted in more severe disease symptoms and reduced plant health. Importantly, Fang, Zhang et al. [49] found that no single alfalfa variety was resistant to both pathogens under co-infection, highlighting the complexity of breeding for disease resistance in the presence of multiple pathogens, proving the importance of the discovery of cultivar ‘Elephant’ and its resistance to both single and dual fungal infections on garlic. Lamichhane and Venturi [50] and Abdullah, Moffat et al. [34] noted that pathogen–pathogen interactions can lead to more severe disease symptoms than those caused by single infections and can significantly alter host responses. Similarly, Susi, Barrès et al. [35] observed that co-infection with two strains of the fungal pathogen *Podosphaera plantaginis* in *Plantago lanceolata* significantly elevated disease prevalence, as compared to infections involving only a single strain. These observations are particularly relevant to our garlic study, where co-inoculation with *F. proliferatum* and *S. cepivora* resulted in significantly increased disease severity on garlic cultivars. The observed transition from competitive co-existence *in vitro* to synergistic pathogenicity *in planta* is consistent with the dynamic nature of fungal interactions and confirms the validity of the experimental findings. In the study of Marchetto and Power [51], the effects of co-infection timing on barley infected with barley yellow dwarf virus and barley stripe mosaic virus were examined, finding that simultaneous co-infections were significantly more damaging to the host than sequential co-infections, leading to greater reductions in seed production. This partially mirrors our findings in garlic, where despite the fact that sequential infections were not studied, the simultaneous co-inoculation with *F. proliferatum* and *S. cepivora* significantly increased disease severity on garlic compared to single infections.

The ‘Elephant’ cultivar’s notable resistance, even under co-infection conditions, distinguished it from other cultivars, which demonstrated significant susceptibility under similar conditions. This robust resistance is presumably underpinned by complex defense mechanisms, potentially facilitated by specific genetic traits that manifest structural or biochemical barriers to pathogenic infection. These observations corroborate the observations of Tollenaere, Susi et al. [33], which underscore the significance of genetic diversity within plant populations in imparting resistance to a multitude of pathogens. The steadfast resistance displayed in our study by the ‘Elephant’ cultivar to both single and concurrent infections in controlled environments necessitates further validation within field settings and a comprehensive analysis of its genetic framework. Studying the genetic foundations of the resistance exhibited by ‘Elephant’ would represent a significant advancement in the development of disease management strategies of Alliums. Through detailed genetic characterization, breeders can cultivate new garlic varieties endowed with similar resilient traits, thereby augmenting crop resilience against diverse and combined pathogen threats and promoting sustainable agricultural methodologies.

## 5. Conclusions

This study explored the co-occurrence of garlic pathogens *F. proliferatum* and *S. cepivora* in garlic fields, the dynamics of the pathogen–pathogen interaction, and the resistance profiles of eleven garlic cultivars under *in planta* conditions. Dual culture assays revealed that *F. proliferatum* and *S. cepivora* exhibit competitive coexistence, highlighting a new way to study the dynamics of multiple pathogenic fungi interactions due to multiple infections on garlic.

Resistance screening among cultivars revealed considerable variability in the resistance profiles among the tested cultivars in individual inoculations, ranging from high susceptibility to partial and high resistance. Notably, simultaneous inoculations of *F. proliferatum* and *S. cepivora* eradicated any partial resistance observed under individual inoculation conditions in all cultivars except for ‘Elephant’, confirming that the pathogen–pathogen interaction between the two fungi is competitive co-existence, as their combined presence on the garlic cloves’ hosts resulted in more rapid infections along with more severe symptoms. Additionally, the ‘Elephant’ cultivar demonstrated not only resistance to individual infections by *F. proliferatum* and *S. cepivora* but also to their combined infection. This unique and novel resistance profile of ‘Elephant’ suggests that it possesses genetic traits conferring a broad spectrum of resistance mechanisms, potentially making it an invaluable resource for future breeding programs.

To build on these findings, future research should include field trials to help ascertain the practical applicability of the observed resistances under natural conditions and assess the influence of environmental factors on disease severity and cultivar resistance to these pathogens. Utilizing advanced genetic analyses could identify and characterize the specific genes or genetic traits responsible for resistance in the ‘Elephant’ cultivar and other partially resistant cultivars under single inoculations. This could aid in the development of genetically engineered garlic cultivars with enhanced disease resistance.

## Figures and Tables

**Table 1:** Summary of the experimental designs used in this study, including the number of independent experiments, replicates, and statistical analyses used.

Experiment	Number of Experiments	Number of Replicates	Samples Per Replicate	Statistical Analysis
Dual culture experiment of *F. proliferatum* and *S. cepivora* on Petri dishes	3	4	Each replicate consisted of one dual-culture/single-culture petri dish	One-way ANOVA, descriptive statistics, and effect size (η^2^)
Single and dual inoculations of *F. proliferatum* and *S. cepivora* on garlic	3	5	Each replicate included 5 garlic cloves	One-way ANOVA, descriptive statistics, effect size (η^2^), and Tukey post-hoc test

## Data Availability

The data presented in this study are available on request from the corresponding author.

## Supplementary Materials

The following supporting information can be downloaded at: https://www.mdpi.com/article/10.3390/plants14030440/s1, Figure S1: Phylogenetic tree constructed using the Maximum Likelihood (ML) method based on the ITS region sequences of *Fusarium* spp. The tree was generated with MEGA software, and the branch labels represent genetic distances (substitutions per site) between sequences. The isolate “*Fusarium proliferatum* isolate (ITS1-2|F.sp T42-079)” is highlighted in red and shows its evolutionary relationship with closely related *Fusarium* species. The sequences are labeled with GenBank accession numbers and species names; Figure S2: Phylogenetic tree constructed using the Maximum Likelihood (ML) method based on the ITS region sequences of *Stromatinia* spp. (*Sclerotium* spp.). The tree was generated with MEGA software, and the branch labels represent genetic distances (substitutions per site) between sequences. The isolate “*Stromatinia cepivora* isolate (ITS1-2|S.c T42-079)” is highlighted in red and shows its evolutionary relationship with closely related *Fusarium* species. The sequences are labeled with GenBank accession numbers and species names; Table S1: ANOVA analyses results of the growth inhibition of *F. proliferatum* and *S. cepivora* at Day 8 and Day 14; Table S2: ANOVA effect sizes analyses results of the growth inhibition of *F. proliferatum* and *S. cepivora* at Day 8 and Day 14; Table S3: ANOVA analyses results of the radial growth of *F. proliferatum* and *S. cepivora* when co-cultured at Day 8 and Day 14; Table S4: ANOVA effect sizes analyses results of the radial growth of *F. proliferatum* and *S. cepivora* when co-cultured at Day 8 and Day 14; Table S5: ANOVA analyses results of single and dual inoculations of *F. proliferatum* and *S. cepivora*; Table S6: ANOVA effect sizes analyses results of single and dual inoculations of *F. proliferatum* and *S. cepivora.*
