# Supplementary material for: Co-Occurrence of Stromatinia cepivora and Fusarium proliferatum Fungi on Garlic: In Vitro Investigation of Pathogen–Pathogen Interactions and In Planta Screening for Resistance of Garlic Cultivars
Source: Plants (Basel). 2025 Feb 2;14(3):440. doi: 10.3390/plants14030440 (PMC11820350; doi:10.3390/plants14030440)
Supplement: Supplementary file 1 [file plants-14-00440-s001.zip › ANOVA result tables.pdf]

Table S1. ANOVA analyses results of the growth inhibition of *F. proliferatum* and *S. cepivora* at Day 8 and Day 14.

**ANOVA Growth inhibition (%)**					
**Day 8**	**Sum of Squares**	**df**	**Mean Square**	**F**	**Sig.**
Between Groups	21,319	1	21,319	6,732	0,017
Within Groups	69,671	22	3,167		
Total	90,990	23			
**Day 14**	**Sum of Squares**	**df**	**Mean Square**	**F**	**Sig.**
Between Groups	62,921	1	62,921	33,366	< 0,001
Within Groups	41,487	22	1,886		
Total	104,408	23			

Table S2. ANOVA effect sizes analyses results of the growth inhibition of *F. proliferatum* and *S. cepivora* at Day 8 and Day 14.

**ANOVA Effect Sizes (Growth inhibition)**				
		**Point Estimate**	**95% Confidence Interval**	
			**Lower**	**Upper**
**Day 8**	**Eta-squared**	0,234	0,007	0,476
	**Epsilon-squared**	0,200	-0,038	0,452
	**Omega-squared Fixed-effect**	0,193	-0,036	0,442
	**Omega-squared Random-effect**	0,193	-0,036	0,442
		**Point Estimate**	**95% Confidence Interval**	
			**Lower**	**Upper**
**Day 14**	**Eta-squared**	0,603	0,292	0,743
	**Epsilon-squared**	0,585	0,260	0,731
	**Omega-squared Fixed-effect**	0,574	0,252	0,723
	**Omega-squared Random-effect**	0,574	0,252	0,723
Eta-squared and Epsilon-squared are estimated based on the fixed-effect model.<br>Negative but less biased estimates are retained, not rounded to zero.				

Table S3. ANOVA analyses results of the radial growth of *F. proliferatum* and *S. cepivora* when co-cultured at Day 8 and Day 14.

**ANOVA Radial growth (cm)**					
**Day 8**	**Sum of Squares**	**df**	**Mean Square**	**F**	**Sig.**
Between Groups	0,327	1	0,327	29,585	< 0,001
Within Groups	0,243	22	0,011		
Total	0,570	23			
**Day 14**	**Sum of Squares**	**df**	**Mean Square**	**F**	**< 0,001**
Between Groups	0,327	1	0,327	29,585	< 0,001
Within Groups	0,243	22	0,011		
Total	0,570	23			

Table S4. ANOVA effect sizes analyses results of the radial growth of *F. proliferatum* and *S. cepivora* when co-cultured at Day 8 and Day 14.

ANOVA Effect Sizes (Radial growth)				
		Point Estimate	95% Confidence Interval	
			Lower	Upper
**Day 8**	**Eta-squared**	0,574	0,256	0,724
	**Epsilon-squared**	0,554	0,222	0,711
	**Omega-squared Fixed-effect**	0,544	0,215	0,702
	**Omega-squared Random-effect**	0,544	0,215	0,702
		**Point Estimate**	**95% Confidence Interval**	
			**Lower**	**Upper**
**Day 14**	**Eta-squared**	0,574	0,256	0,724
	**Epsilon-squared**	0,554	0,222	0,711
	**Omega-squared Fixed-effect**	0,544	0,215	0,702
	**Omega-squared Random-effect**	0,544	0,215	0,702
Eta-squared and Epsilon-squared are estimated based on the fixed-effect model.				

Table S5. ANOVA analyses results of single and dual inoculations of *F. proliferatum* and *S. cepivora.*

ANOVA Disease incidence (%)					
***F. proliferatum* single inoculations**	**Sum of Squares**	**df**	**Mean Square**	**F**	**Sig.**
Between Groups	74,754	10	7,475	76,727	< 0,001
Within Groups	79,307	814	0,097		
Total	154,061	824			
***S. cepivora* single inoculations**	**Sum of Squares**	**df**	**Mean Square**	**F**	**Sig.**
Between Groups	61,265	10	6,127	77,182	< 0,001
Within Groups	64,613	814	0,079		
**Dual inoculations**	**Sum of Squares**	**df**	**Mean Square**	**F**	**Sig.**
Between Groups	64,996	10	6,500	301,981	< 0,001
Within Groups	17,520	814	0,022		

Table S6. ANOVA effect sizes analyses results of single and dual inoculations of *F. proliferatum* and *S. cepivora.*

ANOVA Effect Sizes (Disease incidence)				
		**Point Estimate**	**95% Confidence Interval**	
			**Lower**	**Upper**
***F. proliferatum* single inoculations**	**Eta-squared**	0,485	0,434	0,520
	**Epsilon-squared**	0,479	0,427	0,514
	**Omega-squared Fixed-effect**	0,479	0,427	0,514
	**Omega-squared Random-effect**	0,084	0,069	0,096
		**Point Estimate**	**95% Confidence Interval**	
			**Lower**	**Upper**

***S. cepivora* single inoculations**	**Eta-squared**	0,487	0,435	0,522
	**Epsilon-squared**	0,480	0,428	0,516
	**Omega-squared Fixed-effect**	0,480	0,428	0,516
	**Omega-squared Random-effect**	0,085	0,070	0,096
		**Point Estimate**	**95% Confidence Interval**	
			**Lower**	**Upper**
**Dual inoculations**	**Eta-squared**	0,788	0,763	0,804
	**Epsilon-squared**	0,785	0,760	0,802
	**Omega-squared Fixed-effect**	0,785	0,760	0,801
	**Omega-squared Random-effect**	0,267	0,240	0,288
Eta-squared and Epsilon-squared are estimated based on the fixed-effect model.				
