# Supplementary material for: Co-Occurrence of Stromatinia cepivora and Fusarium proliferatum Fungi on Garlic: In Vitro Investigation of Pathogen–Pathogen Interactions and In Planta Screening for Resistance of Garlic Cultivars
Source: Plants (Basel). 2025 Feb 2;14(3):440. doi: 10.3390/plants14030440 (PMC11820350; doi:10.3390/plants14030440)
Supplement: Supplementary file 1 [file plants-14-00440-s001.zip › Phylogenetic trees.pdf]

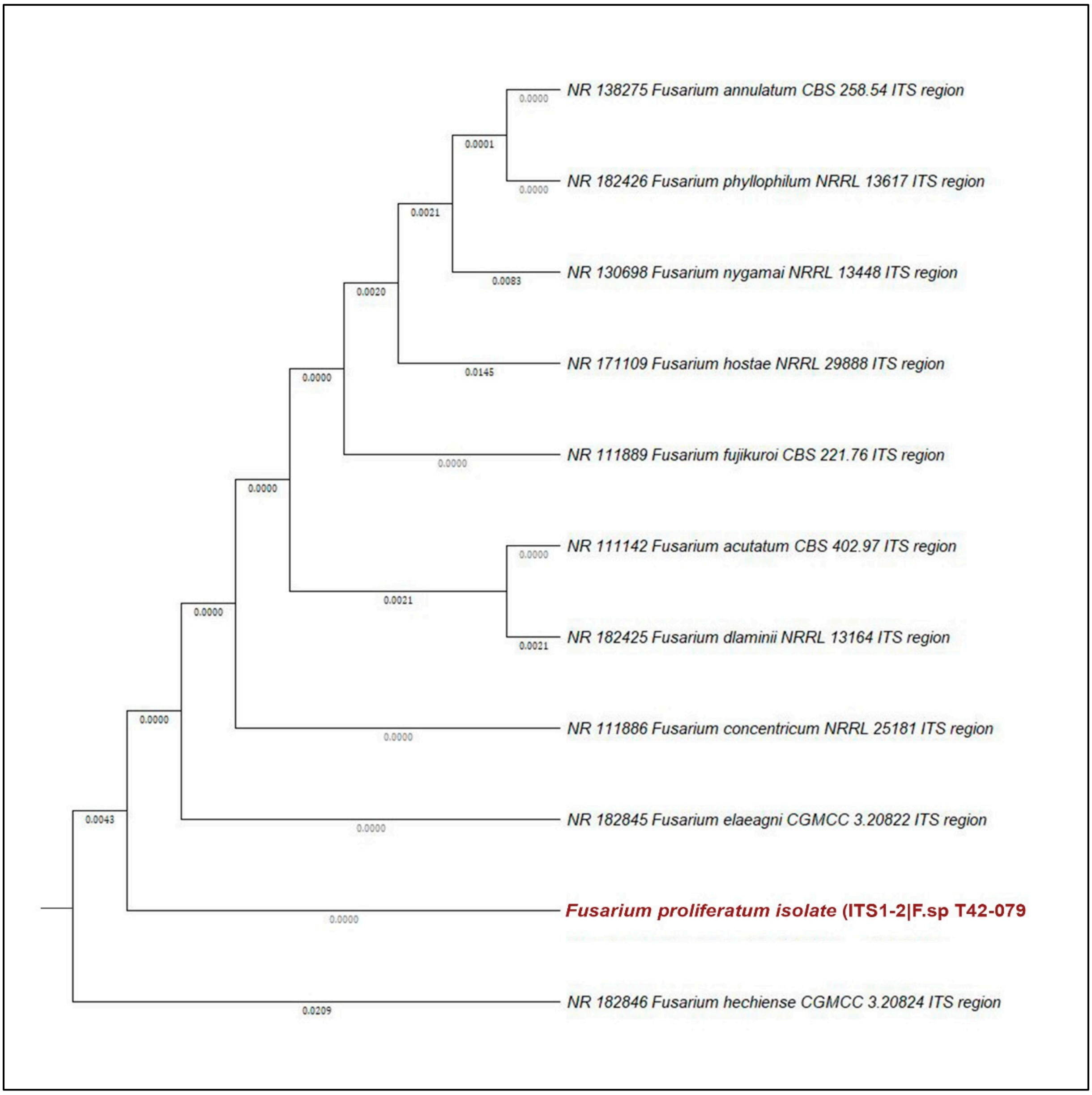

Figure S1. Phylogenetic tree constructed using the Maximum Likelihood (ML) method based on the ITS region sequences of *Fusarium* spp. The tree was generated with MEGA software, and the branch labels represent genetic distances (substitutions per site) between sequences. The isolate "*Fusarium proliferatum* isolate (ITS1-2|F.sp T42-079)" is highlighted in red and shows its evolutionary relationship with closely related *Fusarium* species. The sequences are labeled with GenBank accession numbers and species names.

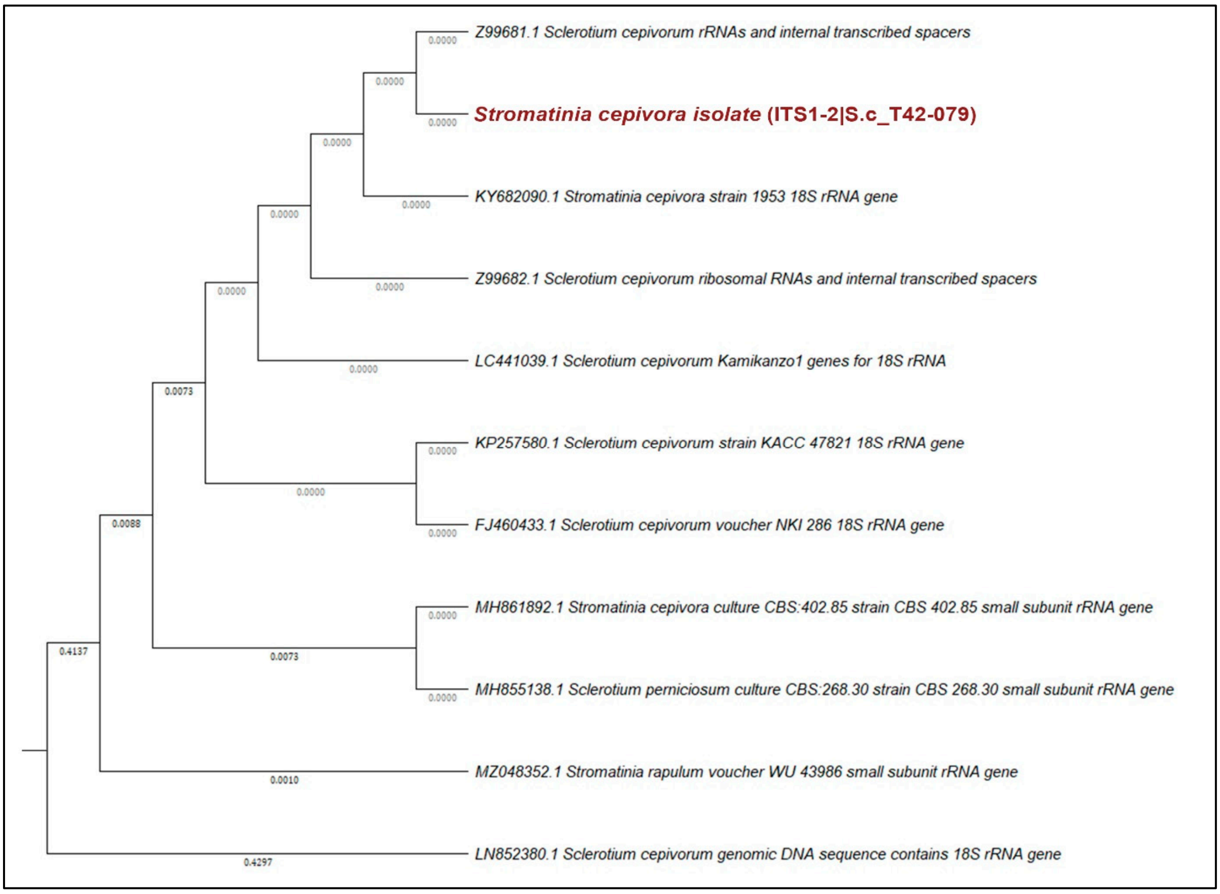

Figure S2. Phylogenetic tree constructed using the Maximum Likelihood (ML) method based on the ITS region sequences of *Stromatinia* spp. (*Sclerotium* spp.). The tree was generated with MEGA software, and the branch labels represent genetic distances (substitutions per site) between sequences. The isolate "*Stromatinia cepivora* isolate (ITS1-2|S.c T42-079)" is highlighted in red and shows its evolutionary relationship with closely related *Fusarium* species. The sequences are labeled with GenBank accession numbers and species names
